# Health care providers' perceptions of family participation in essential care in the intensive care unit: A qualitative study

**DOI:** 10.1111/nicc.13188

**Published:** 2024-10-21

**Authors:** Boukje M. Dijkstra, Lisette Schoonhoven, Karin M. Felten‐Barentsz, Margriet J. M. van der Valk, Johannes G. van der Hoeven, Lilian C. M. Vloet

**Affiliations:** ^1^ Research Department Emergency and Critical Care HAN University of Applied Sciences, School of Health Studies Nijmegen The Netherlands; ^2^ Intensive Care Unit Radboud University Medical Center Nijmegen The Netherlands; ^3^ Nursing Science, Julius Center for Health Sciences and Primary Care University Medical Center Utrecht, Utrecht University Utrecht The Netherlands; ^4^ School of Health Sciences, Faculty of Environmental and Life Sciences University of Southampton Southampton UK; ^5^ Department of Rehabilitation—Physical Therapy, Radboud Institute for Health Sciences Radboud University Medical Center Nijmegen The Netherlands; ^6^ IQ healthcare, Radboud Institute for Health Sciences Radboud University Medical Center Nijmegen The Netherlands; ^7^ Foundation Family and patient Centered Intensive Care Alkmaar The Netherlands

**Keywords:** essential care, family participation, intensive care unit, nursing, relatives

## Abstract

**Background:**

Family participation in essential care may benefit patients and relatives.

**Aim:**

To examine the needs, perceptions and preferences of health care providers about family participation in essential care in the adult intensive care unit.

**Study design:**

A qualitative descriptive study using inductive thematic analysis. Three focus group interviews with a total of 30 intensive care unit health care providers, consisting of 20 critical care nurses, one nursing assistant, five physicians, three physical therapists and one speech therapist working in three Dutch intensive care units.

**Results:**

One overarching theme, *balancing interests*, and four main themes emerged: *looking after the patient's interests*, *taking the relatives' perspective into account*, *looking after interests of intensive care unit health care providers* and *conditions for family participation.* The first theme, *looking after the patient's interests*, included three sub‐themes: insecurity about patient's wishes and needs, patient safety concerns and potential benefits for the patient. The second theme, *taking the relatives' perspective into account*, was also characterized by three sub‐themes: concerns about the relatives' possible burden, potential benefits for the relative and the relationship between patient and relative. The third theme, *looking after interests of intensive care unit health care providers*, included three sub‐themes: attitude towards family participation in essential care, differing perceptions of essential care and concerns about intensive care unit health care provider's burden. The last theme, *conditions for family participation*, included two sub‐themes: establishing a relationship and considering family participation in essential care as a process.

**Conclusions:**

Health care providers' perceptions and preferences regarding family participation in essential care in the intensive care unit are summarized in the overarching theme *balancing interests*. This overarching theme also reflects the needs and perceptions of patients and relatives.

**Relevance to Clinical Practice:**

These findings may support critical care nurses and other health care providers when encouraging family participation in essential care.

**What is known about the topic**

- Family participation in essential care activities may be beneficial for relatives and patients.

**What this paper adds**

- The overarching theme, *balancing interests*, characterizes health care providers' perceptions and preferences regarding family participation in essential care activities in the intensive care unit.
- Health care providers feel responsible for the patient's, relatives' and their own interests. These interests vary and may even conflict sometimes, requiring them to balance these interests.
- *Balancing interests* is also reflected in the patient's and relatives' needs and perceptions.

## 1 INTRODUCTION

Patients and relatives often find admission to the intensive care unit (ICU) stressful, and patients report long‐term physical, cognitive or mental health impairments afterwards, known as ‘post‐intensive care syndrome’ (PICS).^1^, ^2^ Also, relatives are at risk of developing anxiety, depressive and/or post‐traumatic stress symptoms as a result of the patient's ICU stay, described as PICS‐Family (PICS‐F).^3^, ^4^

## 2 BACKGROUND

In a recent review, the use of multifaceted interventions was proposed to reduce relatives' burden and to influence their mental health outcomes.^5^ Family participation in essential care activities may be considered as such an intervention. This relates to a broader concept, family involvement, that can be considered as a continuum ranging from passive, such as ‘presence’, to active ‘contribution to care’.^6^ Family participation also fits well with current developments in ICU care with increasing attention for the provision of patient and family‐centred care (PFCC). PFCC is an ‘approach to the planning, delivery, and evaluation of healthcare, grounded in mutually beneficial partnerships among healthcare providers (HCPs), patients, and families’.^7^ Essential care includes communication, amusement/distraction, comfort, personal care, breathing exercises, mobilization and nutrition. Examples of essential care activities that relatives can participate in are combing hair, application of body lotion, assisting with bed bathing or with changing the patient's position in bed.^8^, ^9^, ^10^ These activities also depend on the individual patient's situation, requiring a personalized approach.

In a previous integrative review, we investigated patients', relatives' and ICU HCPs' needs, perceptions, preferences and capacities regarding family participation in essential care activities.^11^ For the ICU HCPs, eight themes emerged. Generally, most ICU HCPs had a positive attitude towards family participation, while preferences for care activities varied. They mentioned potential beneficial effects on patient safety and quality of care and the opportunity for building a relationship with relatives. Some, however, perceived stress because of the presence of or interaction with relatives. They experienced a loss of control with relatives influencing their working time and space. Several ICU HCPs expressed the need for education, training and guidelines to address safety concerns. High patient acuity influenced ICU HCPs' willingness to enable family participation. Lastly, several organizational conditions were considered to have either a positive or negative influence on family participation.

To further explore these perceptions, and especially the needs, preferences and capacities of HCPs regarding family participation in essential care, and a focus on possible influencing factors, we performed a qualitative study using focus group interviews.

## 3 AIM

The primary aim of this study was to examine the needs, perceptions, preferences and capacities according to HCPs about family participation in essential care in the adult ICU. The secondary aim was to identify factors that may affect family participation.

## 4 DESIGN AND METHODS

### 4.1 Design

We performed a qualitative descriptive study using inductive thematic analysis. The study adheres to the Consolidated Criteria for Reporting Qualitative research checklist.^12^

### 4.2 Setting and sample

The study was conducted with ICU HCPs from three ICUs, one general, one teaching and one university hospital in the Netherlands. One ICU had single patient rooms with an open visiting policy, the other two ICUs had both single rooms and multibed rooms and maintained limited visiting hours in the afternoon and evening. Participants were ICU nurses, nursing assistants, physicians, physical therapists and speech therapists working in one of the three ICUs.

### 4.3 Data collection tools and methods

We performed a qualitative descriptive study using inductive thematic analysis. The study adheres to the Consolidated Criteria for Reporting Qualitative research checklist.^12^

Three focus group interviews were conducted between November 2017 and February 2018. One focus group interview was carried out by the primary researcher (BD). She is an ICU nurse with a master's degree in nursing science. Two were conducted by a researcher (AR). She is a lecturer in nursing with a master's degree in nursing science. Both focus group leaders have extensive experience in leading focus group interviews. The semi‐structured focus group interviews were conducted with the use of an interview guide. The interview guide was based on existing literature, expert opinion and the research team members' experience with family participation in essential care activities (see Table 1).

**TABLE 1:** Interview guide to intensive care unit health care provider's experience with family participation in essential care activities.

Opening question: What are your experiences with family participation in essential care in the ICU?
What are your needs regarding family participation?
What are possible care activities that relatives can participate in?
What contextual factors play a role? For example: size of patient room, privacy, visiting hours, time, colleagues and managers?
What are facilitating factors for family participation in essential care activities?
What are impeding factors for family participation in essential care activities?

All focus group interviews were audio recorded and transcribed verbatim by the researchers (BD, KFB, MvdV). After the first focus group interview, the audio recording was assessed by two fellow researchers (KFB, MvdV), and no modifications in the interview guide or conduct of the focus group interview were considered necessary. The primary researcher (BD) made field notes during and after the focus group interviews. Data collection ended when no new findings were identified, as discussed and agreed upon by two researchers (BD, LV), and data saturation was reached.

### 4.4 Data analysis

All interview transcripts were imported in ATLAS.ti (version 9) software for analysis of qualitative data. One focus group interview transcript was analysed independently by two researchers (BD, LV). Findings were compared and differences were discussed until consensus was reached. We performed an inductive thematic analysis, following Braun and Clark.^13^ The two researchers (BD, LV) independently read the entire transcript from the first focus group interview and assigned codes. These codes were reviewed and discussed, followed by one researcher (BD) assigning codes to the remaining two focus group interview transcripts. The derived codes were then reviewed and discussed by the two researchers, and themes were deduced from these codes. Disagreements were discussed with all researchers until consensus was reached. The identified themes were reviewed and discussed by all researchers until consensus was reached. The derived (sub)themes and relevant quotes were translated into English by the primary researcher (BD) and approved by all fellow researchers.

### 4.5 Trustworthiness

To ensure trustworthiness, the following criteria were applied: credibility, transferability, dependability and confirmability.^14^ Credibility was established with prolonged engagement and sufficient time for data collection to gain an in‐depth understanding of family participation in essential ICU care from the ICU HCP's perspective.^15^ The primary researcher (BD) tried to elicit their experiences to uncover needs, perceptions and influencing factors.^15^ Credibility was enhanced further with triangulation through the selection of ICU HCPs from different hospitals with various professions, independent data analysis with two researchers and the use of field notes.^16^ In addition, a diverse research team was deployed, consisting of researchers with backgrounds in (critical care) nursing, critical care medicine, nursing science and physical therapy.

To ensure transferability, a detailed ‘thick description’ was provided of the study participants, setting and the research process.^17^ To establish dependability and confirmability, throughout the research process notes were taken to enable replication.^15^

## 5 ETHICAL AND RESEARCH APPROVALS

The study was approved by the Research Ethics Committee (CMO 2017‐3635) and the Hospital Ethics Committees of the participating ICUs subsequently and complied with the Declaration of Helsinki. Potential participants were provided with verbal and written information regarding the study. Written informed consent was obtained from all participants after expressing willingness to participate. Obtained data were stored on a network disk anonymously, for research purposes specifically, and secured by the HAN University of Applied Sciences. The disk can only be accessed by authorized persons. HAN University of Applied Sciences has set rules in this regard and has its own Ethics Committee.

## 6 RESULTS

Three focus group interviews with 30 ICU HCPs, consisting of 20 ICU nurses, one nursing assistant, five ICU physicians, three physical therapists and one speech therapist working in the three ICUs, were performed. Most participants were female. The first focus group in the university hospital consisted of nine participants, the second in the teaching hospital of eight participants and the third in the general hospital of 13 participants. Demographics of the study participants are presented in Table 2. Focus group interviews lasted between 54 and 74 min.

**TABLE 2:** Demographics of study participants.

Profession	Gender	Hospital	% gender/ profession within focus group	Focus group no.	% profession within focus group	% profession in all focus groups
ICU nurse	Female	University	50	1	66.7	60
ICU nurse	Male	University	50			
ICU nurse	Female	Teaching	83.3	2	75	
ICU nurse	Male	Teaching	16.7			
ICU nurse	Female	General	66.7	3	46.2	
ICU nurse	Male	General	33.3			
MCU nurse	Female	General	100	3	15.4	6.7
Nursing assistant	Female	University	100	1	11.1	3.3
ICU physician	Female	University	100	1	11.1	16.7
ICU physician	Male	Teaching	100	2	12.5	
ICU physician	Female	General	33.3	3	23.1	
ICU physician	Male	General	66.7			
Physical therapist	Female	University	100	1	11.1	10
Physical therapist	Female	Teaching	100	2	12.5	
Physical therapist	Male	General	100	3	7.7	
Speech therapist	Female	General	100	3	7.7	3.3

**FIGURE 1:**
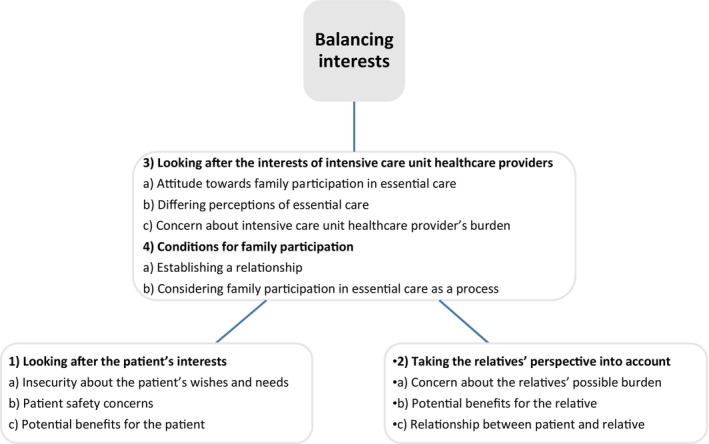
(Overarching) theme(s) and sub‐themes.

Four main themes were identified: *looking after the patient's interests*, *taking the relatives perspective into account*, *looking after the interests of intensive care unit healthcare providers* and *conditions for family participation*. The four main themes could be divided in 11 sub‐themes but finally all themes were summarized in one overarching theme: *balancing interests* (see Figure 1). The following sub‐themes specifically showed the challenges that ICU HCPs face: potential benefits for both patient and relatives, insecurity about the patient's wishes and needs, concerns about patient safety and the relatives' and ICU HCPs' possible burden, ICU HCPs' attitude towards family participation and differing perceptions of essential care, and establishing a relationship. The overarching theme *balancing interests* reflects the responsibility ICU HCPs feel towards the interests of patients, relatives and themselves regarding family participation in essential care activities. The different interests of all involved vary and may even conflict sometimes, where ICU HCPs must balance these interests and adjust their actions accordingly.

### 6.1 Theme 1: Looking after the patient's interests

ICU HCPs are and feel responsible for the well‐being of their patients and try to meet their personal needs, monitor their safety and try to support relatives in activities that may benefit the patient, converging in *looking after the patient*'s *interests*. This theme was characterized by three sub‐themes: ‘insecurity about the patient's wishes and needs‘, ‘patient safety concerns' and ‘potential benefits for the patient’. These sub‐themes are associated with the patient's needs and influence the way ICU HCPs deal with family participation.

#### 6.1.1 Insecurity about the patient's wishes and needs

Several ICU HCPs mentioned insecurity about the patient's wishes and needs often related to the patient's level of consciousness. This is illustrated by the following quotes:

What does this patient want? (ICU nurse 5)

I only invite relatives to participate in care, when patients are awake, that I know they like their relatives to be there. When the patient is sleeping, I consider privacy a bit more sensitive, so then it depends on what we are going to do, whether I invite the relatives. (ICU nurse 14)

In addition, according to ICU HCPs, not all relatives know what the patient's wishes and needs regarding family participation are.

#### 6.1.2 Patient safety concerns

Also, patient safety concerns play a role, and this theme relates to the stability of the patient's situation. ICU equipment can be quite intimidating for relatives and make some essential care activities more challenging or even impossible for relatives to participate in. Some activities require specific skills from ICU nurses and relatives, and this ICU nurse was concerned about the patient's safety, in case of accidental dislocation of a central venous line, while bathing the patient:

I was thinking about legal consequences mostly: if I'm bathing this patient [with a relative], and this relative accidentally removes the central venous line, where does that put me? In the end, I'm responsible…. (ICU nurse 11)

Another example that was mentioned by several ICU HCPs was the risks of dysphagia and aspiration when relatives helped the patient with drinking:

That sip of water, I consider that tricky sometimes … When relatives give the patient a drink …, but they don't know whether the patient can swallow properly, educating relatives is very important. (ICU nurse 10)

The patient's condition, including the patient's level of consciousness, the stability of the patient's situation and the length of the patient's stay in the ICU, was mentioned by several ICU HCPs. When the patient's situation improves and relatives are more used to the situation, it is often easier for them to participate, as the following quote shows:

[Family participation] can be enabled when the patient is more stable. That is often at a later stage. (speech therapist 1)

#### 6.1.3 Potential benefits for the patient

An example that was given by one of the participating ICU nurses was the support a relative can offer when a patient is agitated or delirious and may remove intravenous catheters and feeding tubes. The calming influence some relatives may have is shown in the following quotes:

In case of agitation, that relatives tell [the patient] what time it is, why [the patient] is there, the more often the better, to reduce agitation. (ICU nurse 12)

You can leave the relatives with the patient when the patient is agitated, some relatives have a calming influence. (physical therapist 1)

The speech therapist in one of the focus groups mentioned a possible role for relatives when she had given the patient exercises, to support/enhance communication with the patient or practise swallowing when starting to drink and eat again:

When patients get exercises from us. That relatives practise that with the patient again later.(speech therapist 1)

### 6.2 Theme 2: Taking the relatives' perspective into account

ICU HCPs are concerned with the relatives' well‐being and the possible burden they experience. Therefore, ICU HCPs try to support relatives in activities that may benefit both relatives and the patient, considering their relationship, the type of care activity and relatives' (dis)abilities, converging in *taking the relatives' perspective into account*. This theme was characterized by three sub‐themes: ‘concern about the relatives’ possible burden’, ‘potential benefits for the relative’ and ‘relationship between patient and relative’. These sub‐themes are associated with the relatives' needs and perceptions and influence ICU HCPs in the way they enable family participation.

#### 6.2.1 Concern about the relatives' possible burden

Several ICU HCPs were concerned about the possible burden experienced by relatives, related to the patient's ICU stay. Another ICU nurse mentioned the feelings of powerlessness experienced by relatives, not being able to do much for the patient. She believed that the participation of relatives in helping the patient could be both supportive and stressful, illustrated by the following quote:

Relatives, meanwhile, are helpless, because they can do very little, so it can be very helpful if they can give [the patient] a sip of water or comb their hair or you name it … I also think it's quite a lot you're asking from … relatives. (ICU nurse 12)

You really must anticipate the patient's and relatives’ needs. Some can handle a lot, others hardly anything. (ICU nurse 16)

One of the ICU nurses said that family participation in essential care activities should not feel like an obligation for relatives, it should only be offered as a possibility, as this quote shows:

It should not be mandatory. (ICU nurse 3)

#### 6.2.2 Potential benefits for the relative

Some ICU HCPs recognized the helplessness that many relatives experience and considered the possibility of participating in care activities supportive for them.

It can be very valuable for relatives if they can do something for the patient. (ICU nurse 7)

You see it more often with patients after a longer stay in the ICU, that relatives start helping the patient, often enjoying doing so too. (ICU nurse 14)

Some relatives really feel the need to do something, … sometimes they would like to do something meaningful other than just being there. (physical therapist 3)

#### 6.2.3 Relationship between patient and relative

The relationship between the patient and the relative is also relevant according to several ICU HCPs. One of the ICU nurses mentioned the fact that the relationship between patients and relatives can differ a lot, influencing whether relatives want to participate, and also dependent on the type of (physical) care activity. In the same focus group interview, the case of a mother bathing her sedated adult son was discussed. Several ICU nurses felt uncomfortable with this situation, as this patient was not able to express his wishes.

For example, in case of bathing, I will not ask a friend to participate… only a close relative, a father or mother or a son or daughter. (ICU nurse 4)

### 6.3 Theme 3: Looking after the interests of intensive care unit health care providers

ICU HCPs' attitudes towards family participation vary from positive to hesitant, related to good experiences and possible barriers. Perceptions of essential care varied from physical care activities alone to several options including activities aiming at distraction and comfort. Some ICU nurses had concerns about the possible strain because of additional work in supporting relatives and establishing boundaries, taking needs of all involved into account, resulting in limited space for their own needs. These findings converged in *looking after the interests of intensive care unit healthcare providers*.

This theme was characterized by three sub‐themes: ‘attitude towards family participation in essential care’, ‘differing perceptions of essential care’ and ‘concern about intensive care unit health care provider's burden’. These sub‐themes reflect the ICU HCPs' perceptions and influence the way they enable family participation.

#### 6.3.1 Attitude towards family participation in essential care

From the ICU HCPs' perspective, some had a positive attitude towards family participation and had good experiences, some were a bit hesitant and saw some barriers. One ICU nurse saw a development in the direction of PFCC, with more liberalized visiting policies and ICU HCPs inviting relatives to stay in the room during essential care activities or rounds.

I would like to see us [ICU HCPs] aligned, offering relatives the opportunity to participate. … I see a movement towards involving relatives, also in rounds. (ICU nurse 3)

We have a large team with ICU nurses who say: ‘Nice, come and help.’ But there are also ICU nurses who say: ‘I don't want anyone watching me’ or ‘Isn't that too private, intimate physical care’. So there are all these ifs and buts [regarding physical care], but is that really the biggest part [of family participation], physical care? (ICU nurse 5)

Some ICU nurses experienced difficulties enabling family participation or felt less comfortable, resulting in a more reserved attitude, illustrated by the following quotes:

Sometimes it's difficult, but I think, when relatives indicate that they want to do something, it's nice, working together. (ICU nurse 10)

I think the idea of family participation is great, talking about specific activities, like applying body lotion on feet, those kinds of things. I think that is very nice for relatives. … But I don't think that relatives should be caregivers. (ICU nurse 12)

#### 6.3.2 Differing perceptions of essential care

ICU HCPs had different opinions on essential care activities that could be performed by relatives. Some ICU HCPs appeared more flexible than others. Also, the use of the term essential care led to some discussion. Some ICU nurses were inclined to reduce it to bathing, while others saw wider application, such as reading the newspaper to the patient, applying body lotion or lip balm or combing the patient's hair.

I think we have already created a much better environment than before, and that there is much more than just physical care in the context of family participation.’ (ICU nurse 5)

I usually look for participation in ‘smaller’ activities, so just put on some music with relatives bringing CDs, or the newspaper, so they can read out loud.’ (ICU nurse 6)

It's being with the patient, talking to him, reading the newspaper, I think that that is participation in essential care as well, it is not only bathing, it is so much more. (ICU nurse 10)

Sometimes I let a wife shave her husband, with an electric shaver … applying body lotion, giving a massage, cutting nails, or using the tablet together to watch something or play a game.’ (ICU nurse 17)

In physical therapy, we are really working on involving relatives more … and then it's mainly about moving their feet, hands, fingers, arms. We show them, and tell them what to look out for and then it's up to them whether they want to do it. (physical therapist 1)

#### 6.3.3 Concern about intensive care unit health care provider's burden

Several ICU nurses were concerned about the possible strain they might experience, because of additional tasks in instructing and supporting relatives. They felt they had to take everyone's wishes and needs into account, leaving little room for their own needs. Some felt uncomfortable establishing boundaries with relatives. One of the ICU nurses described the pressure she felt when a patient's mother participated, taking her time, while several colleagues had left the ward for the resuscitation of a patient elsewhere in the hospital. Another ICU nurse shared a similar experience, expressing her concerns when she carried out essential care activities with relatives, and she wanted to take time for this, without feeling rushed. But a shift can be busy at times, and these ICU nurses felt the stress of other tasks that have to be carried out as well:

What I find complicated, when you do this with relatives, you want to take time for this, ease, and sometimes it's busy on the ward, and you think, I really need to do my things now….’ (ICU nurse 8)

I also think it's quite a lot you're asking from us. (ICU nurse 12)

Another ICU nurse speaks of some relatives who do not always have a calming influence on patients, or sometimes even cause agitation:

What I find difficult sometimes is that relatives want to be there 24 hours a day, and they try to wake the patient up, who sometimes needs time to wake up quietly. And I've noticed that this sometimes causes some agitation too, … good guidance and education of relatives is, very important. And we try to do that. But not everyone is always open to it. (ICU nurse 2)

Some ICU nurses were concerned about possible physical consequences, related to the assistance of inexperienced relatives, who have to be taught lifting techniques to reposition the patient, illustrated by the following quote:

In terms of mobilization, I am a bit more reserved about that. They are welcome to help, as long as there are not too many lines and as long as I feel that they can help lift a bit, because I don't want back pain or anything like that. (ICU nurse 16)

### 6.4 Theme 4: Conditions for family participation

ICU HCPs expressed a need for building a relationship between patient, relatives and themselves, facilitating family participation. This relates to regarding family participation as a process, with patients often getting better and relatives feeling more comfortable with the situation, again facilitating family participation, converging in *conditions for family participation*. This theme was characterized by two sub‐themes: ‘establishing a relationship’ and ‘considering family participation in essential care as a process’. The sub‐themes are associated with the ICU HCPs' preferences and capacities.

#### 6.4.1 Establishing a relationship

The need for building a relationship between the patient, relatives and ICU HCPs was expressed several times. When patients have been in the ICU for a longer time, ICU HCPs get to know relatives and the patient better, allowing the building of a relationship. This relationship makes it easier for ICU HCPs to enable family participation. This was illustrated by the following quotes:

Patients who have been with us a bit longer, so at a certain point you get to know the relatives a little, you know a bit about what they would like to do. (ICU nurse 2)

The better you get to know relatives, the easier it is to get them to participate, and it also depends on the relatives, what they want. (ICU nurse 16)

The relationship you build with the relatives then [when enabling family participation], improves. (ICU physician 2)

#### 6.4.2 Considering family participation in essential care as a process

Family participation in essential care activities can be seen as a process, taking into account that the patient's situation is usually improving, and relatives have gotten more used to the situation, making more activities achievable.

The following quotes endorse this sub‐theme:

It's a process, often, the first time, relatives just attend, the next time they dry a hand, the time after that they do more …, it's from nothing to a lot, it's all small steps. (ICU nurse 3)

Participation is a process. (physical therapist 1)

Several ICU nurses also mentioned customization, taking both the individual patient's and relative's situation into account, illustrated by the following quotes:

Family participation should be customised to the patient and relatives. (ICU nurse 3)

You are constantly tuning in with relatives, that remains an ongoing process. (ICU nurse 12)

## 7 DISCUSSION

The aim of this study was to deepen the knowledge on needs, perceptions, preferences and capacities according to ICU HCPs regarding family participation in essential care in the ICU, and to identify factors that may have influenced this. Four main themes were found, summarized in one overarching theme, *balancing interests*. ICU HCPs feel the responsibility to look out for the interests of all involved: *looking after the patient's interests*, *taking the relatives' perspective into account*, *looking after the interests of intensive care unit healthcare providers*. The related sub‐themes reflect everybody's needs and/or perceptions and influence the way ICU HCPs deal with family participation. The last theme, *conditions for family participation*, includes two sub‐themes associated with ICU HCPs' preferences and capacities. All themes come together in one overarching theme, *balancing interests*. ICU HCPs feel responsible for looking after interests of patients, relatives and themselves when enabling family participation in essential care. Aligning the needs and preferences of patients, relatives and ICU HCPs requires good communicative skills and a flexible attitude from ICU HCPs.^18^, ^19^, ^20^ Furthermore, interests differ and may conflict, requiring ICU HCPs to balance these interests and act accordingly. This overarching theme shows similarities with findings from a qualitative study describing family engagement perceptions and practices in ICUs from a global nursing perspective. Family engagement is embedded within nurse–relative interactions, with the location of power circulating between ICU nurses and relatives.^21^ Other studies also describe ICU nurses' efforts to balance conflicting goals regarding the patient's treatment and rehabilitation on one side and compassionate patient‐centred nursing care and comfort on the other side.^22^, ^23^

ICU nurses and other ICU HCPs may also consider customization and personalization, taking the patient's and relative's situation into account, when enabling family participation in essential care.

The sub‐theme ‘*insecurity about the patient's wishes and needs’* was described in a recent study, from the relatives' perspective as well.^24^ Relatives mentioned that it was often difficult to ascertain what the patient's wishes and needs were because of altered levels of consciousness. In the same study, however, most patients reported that they would have appreciated to have their relative participate in care. This was also shown in a study by Garrouste‐Orgeas et al.^25^ where 77.2% patients were in favour of family participation. A different perspective was offered in two more recent studies, with patients letting it happen to them. Patients reported a more passive role, perceiving themselves as receivers of care^26^ or were pragmatic about possible family participation, as they felt unwell or in need of care.^27^ Limited knowledge about patient's needs and perceptions among ICU HCPs and relatives, about family participation in essential care, can be explained by the altered states of consciousness that many ICU patients experience because of sedatives or illness, reducing their ability to express their needs. Future research should aim to gain more insight into the patient's needs, perceptions and preferences regarding family participation, as Mitchell et al. and Dijkstra et al.^11^, ^28^ have previously established. Furthermore, ICU HCPs should discuss possibilities for participation with relatives and when possible with the patient, and align opportunities.

The sub‐theme *‘potential benefits for the patient’* has been illustrated in previous studies, from the patient's perspective, with patients feeling safe and protected in the presence of relatives.^29^, ^30^, ^31^ This was corroborated in our interview study with several patients perceiving their relative participating in care as positive.^24^ Other studies reported the ICU HCP's perspective, generally believing that family participation could benefit patients.^18^, ^28^, ^29^, ^32^, ^33^ Furthermore, family participation could benefit patient safety and quality of care.^34^

The sub‐theme ‘concern about the relatives’ *possible burden*’ was expressed earlier by some patients,^24^ as well as ICU HCPs.^27^, ^28^, ^35^, ^36^, ^37^ Relatives were perceived to be scared or fragile and vulnerable and ICU HCPs feared to increase their stress with family participation. However, this is in contrast with the sub‐theme ‘potential benefits for the relative’, endorsed by two studies indicating that family participation might alleviate stress among relatives.^34^, ^35^, ^37^ Recent studies corroborate this with an increase in satisfaction and decrease in symptoms of anxiety, and post‐traumatic stress among relatives who participated.^38^, ^39^, ^40^ Other studies reported that relatives experienced more comfort, interest, respect, collaboration and support.^37^, ^41^, ^42^ Both sub‐themes are addressed in the suggestion of Xyrichis et al.,^43^ to develop interventions regarding family involvement with closer input from relatives and allowing different levels and kinds of involvement. McAndrew et al.^44^ also endorse ‘potential benefits for the relative’ and suggest a focus for future research on family participation on how to engage relatives and possible outcomes for both patients and relatives.

The sub‐theme ‘differing perceptions of essential care’ relates to findings in a review where preferences for essential care activities varied between and among ICU HCPs, patients and relatives, hindering identification of a uniform list.^11^ The included studies on family participation in essential care activities mostly focused on various physical care activities; some recent studies have included communication and psychosocial care too,^34^, ^45^ reflecting a broader approach of essential care activities. Both differing perceptions and preferences regarding essential care activities hinder implementation of family participation in essential care. This requires a close consideration of perceptions and preferences of ICU HCPs, patients and relatives that should be taken into account before implementation.

Considering the sub‐theme ‘concern about intensive care unit healthcare provider's burden’, several factors have been reported previously as stressful according to ICU HCPs. Some were concerned about patient safety, quality of care and a possible additional burden on relatives.^25^, ^27^, ^28^, ^29^, ^34^, ^35^, ^36^, ^37^, ^46^, ^47^ A survey study among Dutch ICU HCPs revealed that ICU HCPs face relatively high physical, cognitive and emotional demands. Results for ICU nurses showed a low absorption, with absorption being characterized as being focused and absorbed in work. Possible explanations were the demanding work situation, including divided attention, multitasking, requests for information or help, constant alarms, and sometimes hectic situations and life‐threatening circumstances for patients that pose challenges to ICU nurses' workflow.^48^ Also, the presence of and interaction with unpleasant relatives was perceived as stressful by some ICU HCPs.^25^, ^27^, ^36^, ^49^ Some ICU HCPs experienced loss of control and had concerns about relatives controlling their working time and space.^19^, ^27^, ^29^, ^35^, ^36^ These concerns and experiences show parallels with our findings where some ICU HCPs seem to feel uncomfortable establishing boundaries with relatives when enabling family participation. Furthermore, ‘differing perceptions of essential care’ and the finding that some ICU HCPs believe that family participation should be customized to the patient and relatives increase conflicting interests. These conflicting interests may explain why ICU HCPs experience difficulties while enabling family participation in essential care.

The sub‐theme ‘establishing a relationship’ was described previously, as interaction and bridging^43^ and interacting, communication and education.^50^ Relatives experienced improved communication and collaboration and a closer relationship between themselves and ICU nurses,^51^ enabling them to participate.^52^ The relationship between relatives and ICU nurses has received considerable attention in research, justifying shifting focus to organizational, contextual and processual factors that shape the conditions for family involvement.^6^

The other sub‐theme, ‘considering family participation in essential care as a process’, is endorsed in another qualitative study. Relatives and ICU nurses perceived time as essential to develop a relationship establishing rapport and trust with frequent and honest communication.^27^ This process also relates to ‘*development*’, a category synthesized from qualitative studies by Xyrichis et al., that describes the insight that relatives develop regarding the patient's condition and the ICU context, taking relatives' and ICU HCPs' (stressful) perspectives into account.^43^ Wong et al.^52^ described that relatives' willingness to participate in physical care activities varied, and the nature of the care activity and the patient's length of ICU stay influenced this.

### 7.1 Limitations

Although participants were recruited from three different (general, teaching, university) hospitals, inclusion of more hospitals might have increased transferability of the findings.

The focus group interviews were led by two different interviewers, which may have influenced the results. The interviewer in the last focus group interview was present at the first two focus group interviews though, and both interviewers have prepared intensively together, to ensure as much similarity as possible.

## 8 CONCLUSIONS

Critical care health care provider's perceptions, preferences and capacities of family participation in essential care are summarized in an overarching theme: *balancing interests*. Three themes address interests of all involved: *looking after the patient's interests, taking the relatives' perspective into account* and *looking after the interests of intensive care unit healthcare providers* influencing *ICU HCPs* in the way they deal with family participation. The fourth theme, *conditions for family participation*, focusses on establishing a relationship and considering family participation in essential care as a process. Critical care nurses and other health care providers could take these findings into account when stimulating family participation in essential care.

## AUTHOR CONTRIBUTIONS

Boukje Dijkstra: Conceptualization, Methodology, Validation, Formal analysis, Writing—original draft; Lisette Schoonhoven: Formal analysis, Writing—review and editing; Karin Felten‐Barentsz: Formal analysis, Writing—review and editing; Margriet van der Valk: Formal analysis, Writing—review and editing; Johannes van der Hoeven: Formal analysis, Writing—review and editing; Lilian Vloet: Conceptualization, Methodology, Validation, Formal analysis, Writing—review and editing, Funding acquisition. All authors have read and approved the final manuscript.

## FUNDING INFORMATION

This project was funded by the Dutch Research Council (NWO) (RAAK.PUB03.011).

## CONFLICT OF INTEREST STATEMENT

No conflict of interest has been declared by the authors.

## CONSENT FOR PUBLICATION

Written informed consent was obtained from all participants.

## Data Availability

The data that support the findings of this study are available from the corresponding author upon reasonable request.
